# Differential Impact of Polymyxin B Hemadsorption on Long‐Term Mortality in Septic Shock: A Retrospective Analysis of Intra‐Abdominal Versus Extra‐Abdominal Infections

**DOI:** 10.1111/aor.70023

**Published:** 2025-10-08

**Authors:** Tomoki Tanaka, Kazunori Fujino, Yasuyuki Tsujita, Yugo Matsumoto, Mitsuhiro Fujino, Hidemitsu Miyatake, Naoto Mizumura, Junji Shimizu, Takuma Kishimoto, Naoto Shiomi

**Affiliations:** ^1^ Department of Critical and Intensive Care Medicine Shiga University of Medical Science Otsu Japan; ^2^ Shiga University of Medical Science Emergency and Intensive Care Unit Otsu Japan

**Keywords:** abdominal infection, acute blood purification, endotoxin, multiple organ failure, PMX‐HA

## Abstract

**Background:**

The long‐term benefit of polymyxin B hemadsorption (PMX‐HA) in septic shock patients remains unclear and may depend on the site of infection. We evaluated the association between PMX‐HA use and 1‐year mortality in patients with intra‐abdominal infection (IAI) and extra‐abdominal infection (EAI).

**Methods:**

We retrospectively analyzed adult patients with septic shock (Sepsis‐3) admitted to an ICU between January 2017 and July 2023. Patients were categorized as IAI or EAI and further stratified by PMX‐HA use. One‐year mortality was assessed using Kaplan–Meier analysis, multivariable Cox regression (adjusted for age, sex, and SOFA score), and inverse probability of treatment weighting (IPTW), balancing age, sex, SOFA, CRRT use, and with or without surgical and/or radiological interventions.

**Results:**

Among 182 patients (98 IAI, 84 EAI), PMX‐HA was administered to 71 IAI and 32 EAI patients. In the IAI group, PMX‐HA was associated with significantly lower 1‐year mortality (32.3% vs. 59.2%, *p* = 0.005), supported by Cox regression (adjusted HR: 0.485; 95% CI: 0.252–0.935; *p* = 0.031) and IPTW (weighted HR: 0.415; 95% CI: 0.215–0.787; *p* = 0.007). In contrast, in the EAI group, 1‐year mortality was similar between the non‐PMX and PMX groups (44.4% vs. 56.6%, *p* = 0.516), with no significant association in Cox analysis (adjusted HR: 0.790; 95% CI: 0.404–1.543; *p* = 0.49) or IPTW (weighted HR: 0.85; 95% CI: 0.446–1.617; *p* = 0.62).

**Conclusion:**

PMX‐HA was significantly associated with a lower risk of 1‐year mortality in patients with IAI, but not in those with EAI.

## Graphical abstract

In this single‐center retrospective cohort study, the use of PMX‐HA was significantly associated with a reduced risk of 1‐year mortality in patients with septic shock due to intra‐abdominal infection. In contrast, no significant association was observed between PMX‐HA use and 1‐year mortality risk in patients with septic shock caused by extra‐abdominal infection. These findings underscore the significance of infection source in guiding the application of PMX‐HA.

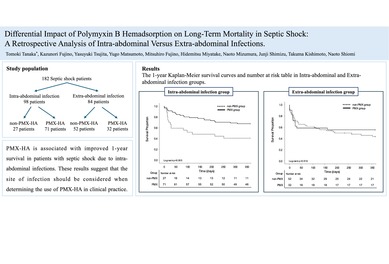

## 1 Introduction

Sepsis remains a condition with a high mortality rate; however, recent updates in clinical guidelines have contributed to a declining trend in short‐term mortality rates [1]. Sepsis is associated with poor short‐term outcomes and unfavorable long‐term prognosis. Given the substantial socioeconomic burden it imposes [2], there is a pressing need to develop therapeutic strategies to improve long‐term outcomes.

Polymyxin B haemadsorption (PMX‐HA) is a blood purification therapy designed to remove endotoxins from the bloodstream [3], and its efficacy has been reported in numerous observational studies [4]. However, while the EUPHAS trial demonstrated an improvement in 28‐day mortality [5], other randomized controlled trials (RCTs), such as the ABDOMIX [6] and EUPHRATES [7], did not show any significant effects on short‐term outcomes, and definitive evidence is yet to be established [8].

One possible reason for uncertainty regarding the short‐term efficacy of PMX‐HA is that its benefits may be limited to specific patient subgroups. Reports from Japan have suggested that PMX‐HA may improve outcomes in elderly patients with high disease severity, as indicated by elevated Sequential Organ Failure Assessment (SOFA) or Acute Physiology and Chronic Health Evaluation II (APACHE II) scores based on findings from a prospective nationwide cohort study [9]. Additionally, a retrospective study utilizing a large diagnostic procedure combination database demonstrated a significant survival benefit of PMX‐HA in moderately severe patients with SOFA scores between 7 and 12 [10]. Furthermore, in patients with septic shock complicated by acute kidney injury requiring continuous renal replacement therapy (CRRT), PMX‐HA was associated with a significant reduction in the 28‐day mortality [11]. In the EUPHRATES trial, patients with septic shock who had a multiple organ dysfunction score greater than 9 and an Endotoxin Activity Assay (EAA) level of ≥ 0.60 were enrolled; however, no significant difference in 28‐day mortality was observed between the PMX‐HA and sham groups (44.5% vs. 43.9%, *p* = 0.97) [7]. Notably, a post hoc analysis limited to patients with EAA levels between 0.60 and 0.89 demonstrated a significant reduction in 28‐day mortality in the PMX‐HA group (26.1% vs. 36.8%, OR: 0.52, 95% CI: 0.27–0.99, *p* = 0.047) [12]. Furthermore, the 1‐year mortality rate was lower in the PMX‐HA group than in the sham group (39.0% vs. 43.3%). These findings suggest that PMX‐HA is a novel therapeutic approach capable of improving long‐term outcomes in patients with septic shock, particularly when appropriate patient selection based on EAA levels is used to optimize its efficacy. Collectively, these reports indicate that precise patient selection is crucial for maximizing the effectiveness of PMX‐HA. Identification of appropriate candidates based on biomarkers, such as EAA, and clinical indicators is essential for defining the target population and achieving better outcomes.

We previously reported that the early initiation of PMX‐HA, particularly in cases triggered by intra‐abdominal infections, may be associated with improved outcomes in patients with sepsis [13]. However, to date, no study has directly compared the efficacy of PMX‐HA between intra‐ and non‐intra‐abdominal sources of infection. In the present study, we conducted a single‐center observational analysis to evaluate the impact of PMX‐HA on the long‐term outcomes of patients with sepsis caused by intra‐ versus extra‐abdominal infections.

## 2 Materials and Methods

### 2.1 Study Population

This single‐center retrospective observational study was conducted at Shiga University of Medical Science Hospital (Shiga, Japan) between January 2017 and July 2023.

The study included patients admitted to the intensive care unit (ICU) during the study period, diagnosed with sepsis or septic shock, and met the Sepsis‐3 criteria [14]. The exclusion criteria were as follows: patients aged 15 years or younger, those who had undergone cardiovascular surgery, and those who had received cardiopulmonary resuscitation. Sites of infection were classified using ICD‐10 codes into the following categories: abdominal, respiratory, skin and soft tissue, urinary tract, bloodstream, and others. Patients with abdominal infections were included in the intra‐abdominal infection (IAI) group, whereas those with infections at other sites were included in the extra‐abdominal infection (EAI) group. This study was approved by the Ethics Committee of Shiga University of Medical Science (Approval No. R2024‐051). Because this was a retrospective study, consent was obtained using the opt‐out method, and the requirement for written informed consent was waived.

### 2.2 Outcomes

The primary outcome of this study was 1‐year mortality. Secondary outcomes included 28‐ and 90‐day mortality and temporal changes in the SOFA score, mean arterial pressure, and catecholamine dosage.

### 2.3 PMX‐HA Procedures

PMX‐HA was performed using an adsorbent column containing 5 mg of polymyxin B per gram of polystyrene fiber (Toraymyxin; Toray Medical Co., Tokyo, Japan). The indications and initiation of PMX‐HA were determined by the attending physician after appropriate fluid resuscitation and catecholamine support. The duration of PMX‐HA was targeted at 24 h and extended up to a maximum of 48 h as needed. Nafamostat mesilate was used as an anticoagulant, and the dose was adjusted to maintain an activated coagulation time of 180–200 s. Blood flow during hemoperfusion was maintained at 80–100 mL/min.

### 2.4 Data Collection

The baseline characteristics of all the participants included age, sex, body mass index, APACHE II score, and SOFA score. For renal function assessment within the SOFA score, urine output was used instead of serum creatinine levels because a large proportion of patients underwent CRRT, which could influence serum creatinine levels. Additional variables collected included ICU length of stay, hospital length of stay, and survival status, all of which were obtained from electronic medical records. Clinical data, such as laboratory results, urine output, blood pressure, and blood gas analyses, were also collected. The doses of vasoactive agents were evaluated using the vasoactive‐inotropic score (VIS) calculated using the following formula [15]:

$$\begin{array}{cc} \mathrm{VIS}\;=\; & \text{dopamine dose}\;\left(\mathrm{\mu g}/\mathrm{kg}/\min\right)+\text{dobutamine dose}\;\left(\mathrm{\mu g}/\mathrm{kg}/\min\right)\\ & +100\times \text{epinephrine dose}\;\left(\mathrm{\mu g}/\mathrm{kg}/\min\right)+10\\ & \times \;\text{milrinone dose}\;\left(\mathrm{\mu g}/\mathrm{kg}/\min\right)+10,000\;\\ & \times \text{vasopressin dose}\;\left(\text{units}/\mathrm{kg}/\min\right)\\ & +100\times \text{norepinephrine dose}\;\left(\mathrm{\mu g}/\mathrm{kg}/\min\right) \end{array}$$

### 2.5 Statistical Analysis

Continuous variables are expressed as medians and interquartile ranges, while categorical variables are presented as counts and percentages. The Mann–Whitney *U* test was used to compare continuous variables between groups, and Fisher's exact test was used for categorical variables. Survival curves were generated using the Kaplan–Meier method, and differences between groups were compared using the log‐rank test. To evaluate the association between PMX‐HA and patient outcomes, a Cox proportional hazards regression analysis was performed. Multivariate analysis was conducted with adjustments for age, sex, and SOFA score as potential confounders, which were preselected based on previously published literature and clinical judgment [16, 17, 18]. To reduce imbalances between the groups, an inverse probability of the treatment weighting (IPTW) model was applied [19]. The propensity score was estimated using logistic regression with PMX‐HA use as the dependent variable and age, sex, SOFA score, CRRT use, and with or without surgical and/or radiological interventions as independent variables. Stabilized inverse probability of treatment weights (IPTW) were then calculated from the estimated scores. Covariate balancing was assessed using absolute standardized mean differences (ASMDs) based on weighted cases. Although ASMDs < 0.1 are generally considered to indicate adequate balance, a threshold of < 0.25 was adopted in accordance with suggestions from some statisticians to avoid the loss of important information [20]. Outcomes were analyzed using a weighted Cox proportional hazards model, and 95% confidence intervals were calculated using robust sandwich variance estimators. All statistical analyses were conducted using SPSS version 22 (SPSS, Chicago, IL, USA) except for the IPTW model, which was applied using EZR (Easy R) (Saitama Medical Center, Jichi Medical University, Saitama, Japan) [21], a graphical user interface for R (R Foundation for Statistical Computing, Vienna, Austria).

## 3 Results

### 3.1 Patient Characteristics

**FIGURE 1:**
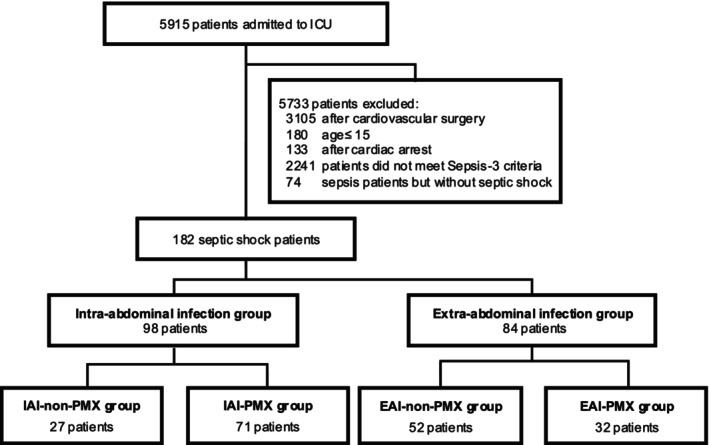
Patient selection. ICU, intensive care unit; PMX, polymyxin B hemadsorption.

Figure 1 shows a flowchart of the patient selection process. During the study period, 5915 patients were admitted to the ICU of our institution. Of these, 3105 were postoperative cardiovascular surgery cases, 180 were patients aged 15 years or younger, 133 were post‐cardiac arrest syndrome cases, and 2241 were non‐sepsis cases. Among the remaining patients, 74 and 182 were diagnosed with sepsis and septic shock, respectively. In total, 182 patients were included in the final analysis. Of the 182 patients, 98 had septic shock due to IAI and 84 had septic shock due to EAI. In the IAI group, 27 patients did not receive PMX‐HA (IAI‐non‐PMX group), whereas 71 patients received PMX‐HA (IAI‐PMX group). In the EAI group, 52 patients did not receive PMX‐HA (EAI‐non‐PMX group), and 32 patients received PMX‐HA (EAI‐PMX group).

Table 1 summarizes the baseline characteristics of patients admitted to the ICU in each group. In both IAI and EAI groups, no significant differences were observed in age, sex, height, or severity scores (APACHE II and SOFA) between the PMX and non‐PMX subgroups. In the IAI group, C‐reactive protein levels were significantly higher than in the PMX group (15.27 vs. 6.18, *p* = 0.031), and a higher proportion of patients underwent emergency surgery or drainage interventions (84.5% vs. 40.7%, *p* = 0.001). The CRRT utilization rate was also significantly higher in the PMX group (97.2% vs. 59.3%, *p* < 0.001). In the EAI group, the central nervous system component of the SOFA score was significantly higher in the PMX group compared to the non‐PMX group (4.0 vs. 2.0, *p* = 0.009; Table S1). Additionally, the proportion of patients receiving CRRT was significantly higher (96.9% vs. 75.0%, *p* = 0.009). [Correction added on 12 February 2026, after first online publication: The preceding sentence has been corrected according to the changes in Table 1.] The proportion of patients who underwent emergency surgical and/or radiological interventions was higher in the PMX group; however, the difference was not statistically significant (34.4% vs. 21.2%, *p* = 0.181). Among patients in the EAI group, respiratory infections were the most common, followed by skin and soft tissue infections (Table S1). The median time from ICU admission to PMX initiation was 3.6 h (2.1–6.3) in the IAI group and 7.3 h (interquartile range: 3.1–16.2) in the EAI group. The duration of PMX treatment was 19.9 h (10.6–25.8) in the EAI group and 23.6 h (14.8–26.3) in the IAI group. All 100 patients who received both CRRT and PMX‐HA had overlapping treatment periods. Simultaneous initiation occurred in 63% of cases. In 34%, PMX‐HA was started after CRRT (median delay: 3 h), while in 3%, CRRT followed PMX‐HA (median delay: 5 h).

**TABLE 1:** Baseline characteristics at ICU admission according to infection site and PMX‐HA treatment.

Characteristics	Intra‐abdominal infection group			Extra‐abdominal infection group		
	Non‐PMX group	PMX group	*p*	Non‐PMX group	PMX group	*p*
Number of patients	27	71		52	32	
Age (years)	72.0 (69.0–78.0)	71.0 (62.0–78.0)	0.336	72.5 (61.0–79.5)	73.5 (66.0–77.8)	0.993
Sex, male, *n* (%)	20 (74.1)	42 (59.2)	0.171	26 (50.0)	21 (65.6)	0.161
BMI (kg/m^2^)	21.3 (18.5–23.1)	22.2 (19.6–25.1)	0.059	22.0 (18.2–24.6)	21.6 (20.1–25.4)	0.724
APACHE II score	24.0 (20.0–27.0)	24.5 (20.0–29.0)	0.519	22.0 (19.0–30.0)	24.5 (17.0–28.0)	0.764
SOFA score	13.0 (8.0–16.0)	12.0 (10.0–14.0)	0.744	12.5 (9.0–14.0)	12.5 (11.0–15.8)	0.102
WBC (×10^3^/μL)	10.4 (6.2–16.3)	7.4 (3.5–11.6)	0.078	12.5 (8.0–20.2)	13.1 (8.0–19.9)	0.949
Platelet count (×10^3^/μL)	171.0 (103.0–218.0)	184.0 (84.0–246.0)	0.41	175.5 (93.8–263.0)	161.5 (66.8–225.3)	0.228
CRP (mg/dL)	6.18 (0.88–16.51)	15.27 (4.24–23.75)	0.031*	14.64 (3.53–22.47)	10.51 (5.40–33.64)	0.607
CRE (mg/dL)	1.43 (0.78–1.78)	1.34 (0.91–2.62)	0.57	1.42 (0.86–2.55)	1.88 (0.94–2.91)	0.324
PCT (ng/ml)	26.0 (1.2–66.1)	14.5 (2.0–56.8)	0.934	13.1 (1.3–31.4)	20.2 (4.5–75.3)	0.13
Hemoglobin (g/dL)	9.8 (8.2–11.1)	10.6 (9.1–12.1)	0.07	10.5 (9.2–12.0)	10.2 (9.3–11.8)	0.967
MAP (mmHg)	62.7 (56.7–73.7)	66.7 (57.7–73.3)	0.815	61.2 (54.1–70.5)	64.7 (59.5–74.9)	0.07
Lactate (mg/dL)	27.0 (24.0–41.0)	27.0 (21.0–43.0)	0.421	26.0 (22.0–43.0)	30.0 (20.0–49.8)	0.694
VIS	21.7 (7.5–28.8)	17.0 (12.4–26.4)	0.858	15.0 (7.3–25.9)	21.3 (14.4–28.7)	0.072
P/F ratio	299.4 (164.0–383.0)	233.2 (160.2–340.0)	0.488	188.9 (102.6–274.2)	139.3 (92.9–318.5)	0.71
Emergency surgical and/or radiological intervention, *n* (%)	11 (40.7)	60 (84.5)	0.001*	11 (21.2)	11 (34.4)	0.181
Pathogenic organisms, *n* (%)						
Gram‐negative bacteria	19 (70.4)	51 (71.8)	0.886	19 (36.5)	17 (53.1)	0.136
Gram‐positive bacteria	2 (7.4)	6 (8.5)	0.866	23 (44.2)	10 (31.2)	0.237
Fungi	0.0	1 (1.4)	0.535	0	0	
COVID‐19	0.0	0		1 (1.9)	1 (3.1)	0.726
Unknown	6 (22.2)	13 (18.3)	0.662	9 (17.3)	4 (12.5)	0.554
CRRT during ICU admission, *n* (%)	16 (59.3)	69 (97.2)	< 0.001*	39 (75.0)	31 (96.9)	0.009*

*Note:* Data are presented as the median (first quartile to third quartile) or number (percentage). [Correction added on 12 February 2026, after first online publication: The unit and values for PCT has been corrected in this version.]

Abbreviations: APACHE II, acute physiology and chronic health evaluation II; BMI, body mass index; CRE, creatinine; CRP, C‐reactive protein; CRRT, continuous renal replacement therapy; ICU, intensive care unit; MAP, mean arterial pressure; P/F ratio, arterial oxygen partial pressure to fraction of inspired oxygen ratio; PCT, procalcitonin; PMX, polymyxin B hemadsorption; SOFA, Sequential Organ Failure Assessment; VIS, vasoactive‐inotropic score; WBC, white blood cells.

* *p* < 0.05.

### 3.2 Outcome

**FIGURE 2:**
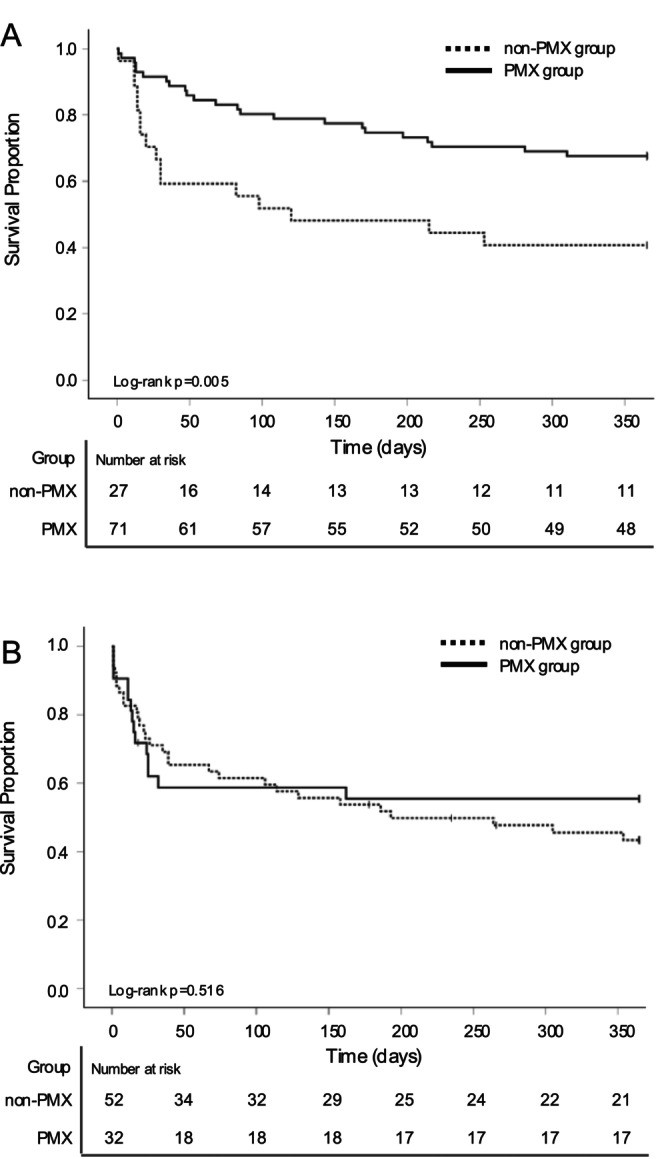
The 1‐year Kaplan–Meier survival curves and number at risk table in intra‐abdominal and extra‐abdominal infection groups. (A) Intra‐abdominal infection group. (B) Extra‐abdominal infection group. Survival rates of patients in the PMX group (solid line) and the non‐PMX group (dashed line). The tick marks indicate censored data. Loss to follow‐up was censored. PMX, polymyxin B hemadsorption.

Figure 2 shows the 1‐year survival curves for each group. In four cases, the 1‐year outcomes could not be confirmed. In the IAI group, the 1‐year mortality rate was significantly lower in the PMX group compared to the non‐PMX group (32.3% vs. 59.2%, *p* = 0.005). Furthermore, both the 28‐ and 90‐day mortality rates were significantly lower in the PMX group (19.7% vs. 40.7%, *p* = 0.022; 27.1% vs. 39.2%, *p* = 0.018, respectively). In contrast, in the EAI group, the 1‐year mortality rate was 44.4% in the PMX group and 56.6% in the non‐PMX group, with no significant difference between the two groups (*p* = 0.516). Similarly, no significant differences were observed in the 28‐ and 90‐day mortality rates between the groups (28.8% vs. 37.5%, *p* = 0.434, and 38.4% vs. 43.8%, *p* = 0.587, respectively). Table 2 presents the results of the Cox regression analyses for each group. In the IAI group, PMX‐HA was significantly associated with a reduced risk of 1‐year mortality in the multivariate analysis (HR: 0.485; 95% CI: 0.252–0.935; *p* = 0.031; Table 2). While the 28‐day mortality (HR: 0.442; 95% CI: 0.143–1.362; *p* = 0.155) and 90‐day mortality (HR: 0.500; 95% CI: 0.223–1.178; *p* = 0.093) showed a non‐significant trend toward lower mortality (Table S2). In contrast, in the EAI group, multivariate analysis showed that PMX‐HA was not significantly associated with a reduced risk of 1‐year mortality (HR: 0.790; 95% CI: 0.404–1.543; *p* = 0.49; Table 2). No significant associations with the risk of mortality were observed for 28‐day mortality (HR: 1.364; 95% CI: 0.613–3.036; *p* = 0.447) or 90‐day mortality (HR: 1.175; 95% CI: 0.571–2.418; *p* = 0.661) either (Table S2). After IPTW, all confounding variables achieved adequate balance in both the IAI and EAI groups, with ASMDs below 0.25. The median (IQR) and maximum stabilized weights were 0.778 (0.757–0.816) and 4.17 in the IAI group, and 0.886 (0.684–1.127) and 2.76 in the EAI group. In the IAI group, PMX‐HA use was significantly associated with a reduced risk of mortality at all time points: the weighted hazard ratio (wHR) for 1‐year mortality was 0.415 (95% CI: 0.215–0.787; *p* = 0.007), for 28‐day mortality 0.302 (95% CI: 0.102–0.900; *p* = 0.03), and for 90‐day mortality 0.395 (95% CI: 0.179–0.736; *p* = 0.02). In contrast, in the EAI group, wHR for 1‐year mortality was 0.85 (95% CI: 0.446–1.617; *p* = 0.62). The wHRs for 28‐day and 90‐day mortality were 1.402 (95% CI: 0.650–3.023; *p* = 0.39) and 1.234 (95% CI: 0.619–2.463; *p* = 0.55), respectively.

**TABLE 2:** Univariate and multivariate Cox regression analyses of PMX‐HA Effect on 1‐year mortality in intra‐abdominal and extra‐abdominal infections.

	Unadjusted		Adjusted	
	HR (95% CI)	*p*	HR (95% CI)	*p*
*Intra‐abdominal infection group*				
Age	1.041 (1.011–1.072)	0.007*	1.038 (1.007–1.071)	0.017*
Sex	1.351 (0.694–2.630)	0.376	1.164 (0.590–2.295)	0.661
SOFA score	0.015 (0.932–1.105)	0.733	1.011 (0.937–1.090)	0.777
PMX‐HA	0.410 (0.216–0.778)	0.006*	0.485 (0.252–0.935)	0.031*
*Extra‐abdominal infection group*				
Age	1.038 (1.010–1.066)	0.007*	1.033 (1.004–1.062)	0.026*
Sex	1.008 (0.552–1.841)	0.978	0.910 (0.489–1.695)	0.767
SOFA score	1.100 (1.005–1.205)	0.038*	1.089 (0.984–1.205)	0.098
PMX‐HA	0.811 (0.428–1.536)	0.52	0.790 (0.404–1.543)	0.49

Abbreviations: CI, confidence interval; HR, hazard ratio; PMX‐HA, polymyxin B hemadsorption.

* *p* < 0.05.

**FIGURE 3:**
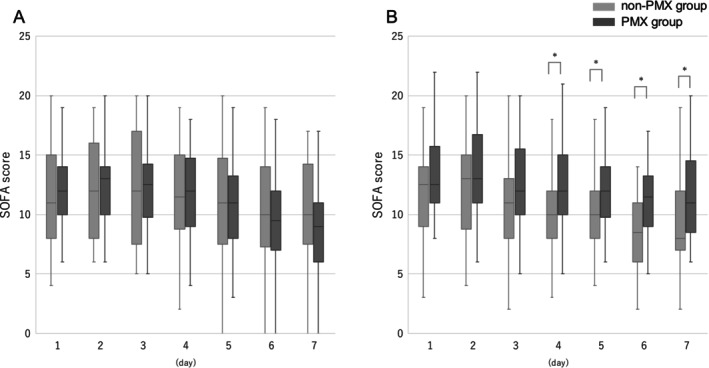
Comparative trends in daily SOFA scores during the first 7 days of ICU admission in intra‐abdominal and extra‐abdominal infection groups. (A) Intra‐abdominal infection group. (B) Extra‐abdominal infection group. Comparison of SOFA scores between patients in the non‐PMX (light gray box) and PMX groups (black box). The centerlines indicate median values, boxtops and bottoms indicate interquartile ranges, and error bars indicate overall ranges. *p < 0.05, when the two groups were compared using the Mann–Whitney *U* test. PMX, polymyxin B hemadsorption; SOFA, Sequential Organ Failure Assessment.

Figure 3 illustrates the temporal changes in SOFA scores after ICU admission in each group. No significant differences were observed among the groups in the IAI group. In contrast, in the EAI group, the PMX group exhibited significantly higher SOFA scores from day 4 to 7. Figure S1 shows the time‐course changes in the individual SOFA components for each group. In the IAI group, no significant differences were observed between the PMX‐HA and non‐PMX‐HA groups for all components. However, in the EAI group, the coagulation component was significantly higher than that in the PMX‐HA group from days 4 to 6.

**FIGURE 4:**
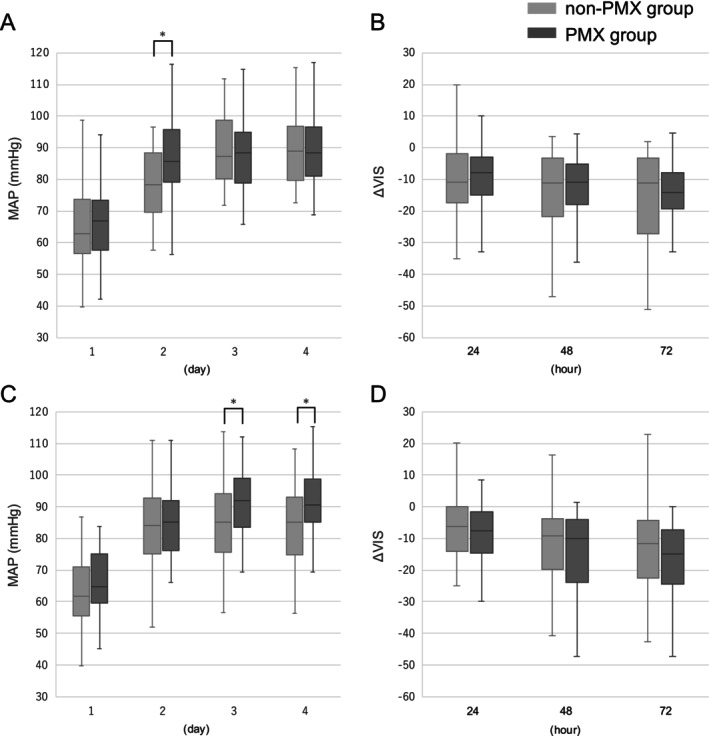
Comparative trends in MAP and ΔVIS in intra‐abdominal and extra‐abdominal infection groups. Boxplots show the time course of mean arterial pressure (MAP) and changes in vasoactive‐inotropic score (ΔVIS) between the non‐PMX group (light gray boxes) and the PMX group (black boxes). (A) MAP during the first 4 days in the intra‐abdominal infection group. (B) ΔVIS at 24, 48, and 72 h in the intra‐abdominal infection group. (C) MAP during the first 4 days in the extra‐abdominal infection group. (D) ΔVIS at 24, 48, and 72 h in the intra‐abdominal infection group. The centerlines within each box represent the median values, boxes indicate the interquartile range (IQR), and whiskers show the full range. Statistical comparisons were performed using the Mann–Whitney *U* test. *p < 0.05, considered statistically significant. MAP, mean arterial pressure; PMX, polymyxin B hemadsorption; VIS, vasoactive‐inotropic score.

Figure 4 depicts the mean arterial pressure (MAP) up to day 4 from ICU admission and the changes in VIS (ΔVIS = VIS at admission—VIS at 24, 48, and 72 h) within the first 72 h. In the IAI group, MAP on day 2 was significantly higher in the PMX group than in the non‐PMX group (85.6 mmHg vs. 78.3 mmHg, *p* = 0.003), while no significant differences were found in ΔVIS between the groups. In the EAI group, the PMX group exhibited significantly higher MAP on day 3 and day 4 compared to the non‐PMX group (92.0 mmHg vs. 85.0 mmHg, *p* = 0.03; 90.6 mmHg vs. 85.0 mmHg, *p* = 0.036, respectively). However, no significant differences were observed in the ΔVIS between the two groups.

## 4 Discussion

In this present study, in cases of intra‐abdominal infection, PMX‐HA was significantly associated with a reduced 1‐year mortality risk, whereas no such association was observed in cases of extra‐abdominal infection. Regarding the progression of SOFA scores, PMX‐HA was not associated with an apparent change in the IAI group. However, in the EAI group, the PMX‐HA group showed significantly higher SOFA scores from days 4 to 7. Furthermore, PMX‐HA significantly increased the mean arterial pressure in both the IAI and EAI groups; however, no significant differences were observed in changes in the VIS compared with the non‐PMX group.

To our knowledge, this is the first study to compare 1‐year outcomes with and without PMX‐HA in patients with intra‐abdominal and extra‐abdominal infections. One of the few previous studies to examine the differential effects of PMX‐HA by infection site, the EUPHAS2 study, investigated patients with septic shock or severe sepsis treated with PMX‐HA. It reported a significant improvement in SOFA scores 72 h after PMX‐HA initiation, but only in patients with intra‐abdominal infections [22]. A subsequent analysis of the EUPHAS2 study further indicated that the therapeutic effect of PMX‐HA was less evident in cases of sepsis or septic shock originating from pulmonary infections than from abdominal sources, with a trend toward higher 28‐day mortality observed in the former group [23]. These findings are consistent with the results of the present study.

One possible explanation for the observed association between PMX‐HA use and better outcomes in the IAI group is the difference in the causative pathogens and the effectiveness of source control. Because PMX‐HA is designed to adsorb endotoxin, it is thought to be particularly effective against gram‐negative bacteria. In this study, the proportion of gram‐negative organisms among the causative pathogens was significantly higher in the IAI group than in the EAI group (71.4% vs. 42.8%), which may help explain the observed association with better outcomes in the IAI group. Another possible contributing factor is the effectiveness of source control, which is one of the most critical components in the management of sepsis. Intra‐abdominal infections often allow for relatively prompt source control through surgical intervention or drainage, potentially resulting in a shorter duration of the infectious burden compared to other infections. In the present study, the proportion of patients who underwent surgical or drainage interventions was higher in the IAI group than in the EAI group (72.4% vs. 26.2%). Moreover, within the IAI group, this proportion was significantly higher in the PMX group compared to the non‐PMX group (84.5% vs. 40.7%). In the EAI group, respiratory infections were the most common (39.3%), and source control for these infections is generally considered to be difficult. In the IPTW analysis including surgical and/or radiological interventions as a covariate, a significant association between PMX‐HA use and reduced mortality risk was observed only in the IAI group. However, the timing and appropriateness of source control could not be evaluated in this study, highlighting the need for further investigation. The association between PMX‐HA use and favorable outcomes may become more evident in patients who undergo appropriate source control.

In this study, PMX‐HA was associated with a lower risk of 1‐year mortality, which may be attributed to its potential immunomodulatory effects. Several studies suggested that PMX‐HA acts as an immunomodulator in patients with sepsis [24, 25, 26, 27, 28]. Secondary infections are a major cause of long‐term mortality in patients with sepsis and are believed to be closely associated with sepsis‐induced immune dysfunction [29]. Our findings suggest that PMX‐HA may have contributed to the improved 1‐year survival by alleviating immune dysfunction, thereby reducing the incidence of secondary infections. However, direct assessment of immune function and detailed analyses of the causes of death were not performed in this study. Further investigation is required to validate this hypothesis. Currently, the EUPHRATES trial [7] remains the only study to have evaluated 1‐year mortality following PMX‐HA therapy. While the findings of the present study contradict those of the EUPHRATES trial, this discrepancy may be attributed to differences in the control populations and methods of PMX‐HA administration, including the timing, duration, and type of anticoagulant used. Further studies are warranted to identify the optimal conditions for PMX‐HA administration with a focus on long‐term outcomes.

Additionally, variations in blood purification strategies including differences in PMX‐HA implementation protocols and the concomitant use of CRRT may have influenced outcomes. In previous RCTs, PMX‐HA has typically been administered in two sessions of 2 h each, with a maximum duration of 2 h per session. However, in the present study, the duration of PMX‐HA was prolonged, with a median treatment time of 20.4 h (IQR: 9.3–25.7). An in vitro study demonstrated that polymyxin B‐immobilized fiber columns retain their endotoxin adsorption capacity for at least 24 h [30]. Furthermore, clinical studies have suggested the potential effectiveness of extended‐duration PMX‐HA in improving patient outcomes [31, 32]. Taken together, the prolonged duration of PMX‐HA observed in the present study may have contributed to the outcomes. In addition, several reports have suggested that early initiation of PMX‐HA may be associated with improved clinical outcomes [13, 33]. In the present study, the median time from ICU admission to the initiation of PMX‐HA was 4.17 h (IQR: 2.25–8.27), suggesting that the timing of initiation may have also influenced the outcomes. Furthermore, previous studies have suggested that PMX‐HA may improve outcomes in septic patients requiring CRRT [11, 34]. In the present study, the rate of CRRT use was higher compared to the previous studies, which may have contributed to the observed results. These findings imply that, in the management of sepsis and subsequent multiple organ failure, the combination of early PMX‐HA initiation and CRRT may be beneficial. This supports the concept of sequential extracorporeal therapy in sepsis, as proposed by De Rosa et al. [35], and warrants further investigation.

Although many studies have reported that PMX‐HA improves SOFA scores [5, 36, 37] and exerts vasopressor effects [7, 12, 38], thereby contributing to better outcomes, these effects were not demonstrated in the present study. In the present study, the SOFA score decreased over time in both PMX‐HA groups, which is consistent with the results of subgroup analyses from the EUPHAS2 study that evaluated changes in SOFA scores among patients with intra‐abdominal or respiratory infections treated with PMX‐DHP‐HA [37]. However, no significant differences were observed in the time course of these scores between the PMX and non‐PMX groups, suggesting that further investigation is needed to determine whether PMX‐HA contributes to the improvement of organ dysfunction. In the EAI group, the significantly higher SOFA scores observed on days 4–7 in the PMX‐HA group may be attributable to the significantly higher coagulation scores in this group. Thrombocytopenia has been reported as a potential adverse effect of PMX‐HA therapy [39], and the findings of our study may reflect this complication. However, in the IAI group, no significant differences were observed in the coagulation components of the SOFA scores between the PMX‐HA and non‐PMX‐HA groups. This may suggest that the risk of PMX‐HA‐associated thrombocytopenia may have been lower in the IAI group, or alternatively, that PMX‐HA may have been associated with overall clinical improvement, including the resolution of coagulopathy, such as disseminated intravascular coagulation. Additionally, in this study, although a difference in the MAP was observed between patients with and without PMX‐HA, both groups showed only modest reductions in the vasopressor dosage. These findings contrast with previous studies that reported hemodynamic improvements with PMX‐HA [7, 12, 38]. One possible explanation is the timing of PMX‐HA initiation relative to the onset of sepsis treatment. Our previous study also demonstrated that a shorter interval between the initiation of the administration of catecholamine and PMX‐HA was associated with a greater reduction in vasopressor requirements [13]. Meanwhile, the widespread adoption of fluid resuscitation and early vasopressor initiation as standard treatments for septic shock has led to earlier treatment initiation. Consequently, the time from treatment initiation to ICU admission and PMX‐HA administration might have been prolonged, potentially diminishing the hemodynamic benefits of PMX‐HA. However, we were unable to collect data to substantiate this hypothesis and further investigation is needed.

Appropriate patient selection is crucial for the effective use of PMX‐HA. Recent randomized controlled trials have demonstrated the efficacy of PMX‐HA when EAA is used as a selection criterion. Further investigations, including the ongoing TIGRIS trial [40], are currently underway. However, EAA measurements are not readily available in all countries and institutions. Under such circumstances, the findings of the present study suggest that PMX‐HA may be a potentially useful treatment option for patients with intra‐abdominal infections, although further validation is required.

This study has several limitations. First, this study was designed as a proof‐of‐concept investigation, and the limited sample size resulted in insufficient statistical power to draw definitive conclusions regarding mortality. Second, this was a single‐center, retrospective, observational study. As our hospital is a tertiary referral center affiliated with a university and primarily admits critically ill patients, there may have been a selection bias, and institutional characteristics could have influenced the results. Although we performed multivariate analyses to adjust for confounding factors, the possibility of unmeasured confounders affecting the outcomes cannot be ruled out. Third, the study population consisted predominantly of Asian patients, limiting the generalizability of the findings to other ethnic groups and precluding the assessment of potential racial differences in response to treatment. Fourth, despite performing weighting using IPTW, some ASMDs exceeded 0.1, which is generally considered indicative of imbalance; therefore, this represents one of the limitations of the present study. On the other hand, based on a previous study suggesting that ASMD values should be below 0.25 to ensure the validity of regression adjustment [20], this degree of imbalance was considered acceptable. Fifth, we were unable to obtain detailed information on the causes of death. Therefore, deaths unrelated to sepsis may have been included in our analysis. Sixth, this study did not evaluate the timing and appropriateness of source control, including emergency surgical and/or radiological interventions, nor did it assess the timing and appropriateness of antimicrobial therapy. Therefore, we cannot fully exclude the possibility that differences in the quality of these interventions influenced the outcomes. Seventh, the proportion of patients who received CRRT differed between groups. Although the use of CRRT was included as a covariate in the IPTW analysis, its potential impact on the study outcome cannot be entirely excluded, highlighting the need for further investigation. Finally, owing to the observational nature of this study, causality could not be established. Further randomized controlled trials focusing on well‐defined target populations are required to determine whether PMX‐HA reduces mortality in patients with septic shock.

## 5 Conclusion

In this single‐center retrospective cohort study, the use of PMX‐HA was significantly associated with a reduced risk of 1‐year mortality in patients with septic shock due to intra‐abdominal infection. In contrast, no significant association was observed between PMX‐HA use and 1‐year mortality risk in patients with septic shock caused by extra‐abdominal infection.

## Author Contributions

T.T. and K.F. contributed to the research idea and study design, and performed the data acquisition and analysis. T.T., J.S., and K.F. wrote the manuscript. Y.T. and N.S. supervised the project. All authors reviewed and approved the final manuscript.

## Conflicts of Interest

The authors declare no conflicts of interest.

## Supporting information


**Figure S1:** aor70023‐sup‐0001‐FigureS1.pdf.

**Table S1:** aor70023‐sup‐0002‐TableS1.xlsx.

**Table S2:** aor70023‐sup‐0003‐TableS2.xlsx.

## Data Availability

The data that support the findings of this study are available from the corresponding author upon reasonable request.
