# Supplementary material for: Differential Impact of Polymyxin B Hemadsorption on Long‐Term Mortality in Septic Shock: A Retrospective Analysis of Intra‐Abdominal Versus Extra‐Abdominal Infections
Source: Artif Organs. 2025 Oct 8;50(2):271–80. doi: 10.1111/aor.70023 (PMC12993260; doi:10.1111/aor.70023)

Supplemental Figure 1. Comparative Trends in Daily SOFA Score Components During the First 7 Days of ICU Admission in the Extra-abdominal and intra- abdominal Infection Group.

(A) Intra-abdominal infection group. (B) Extra-abdominal infection group.

Comparison of SOFA scores between patients in the non-PMX (light gray box) and PMX groups (black box). The centerlines indicate median values, boxtops and bottoms indicate interquartile ranges, and error bars indicate overall ranges.

* $p < 0.05$, when two groups were compared using the Mann–Whitney U test.

SOFA, Sequential Organ Failure Assessment; CNS, central nervous system; PMX, Polymyxin B hemadsorption .

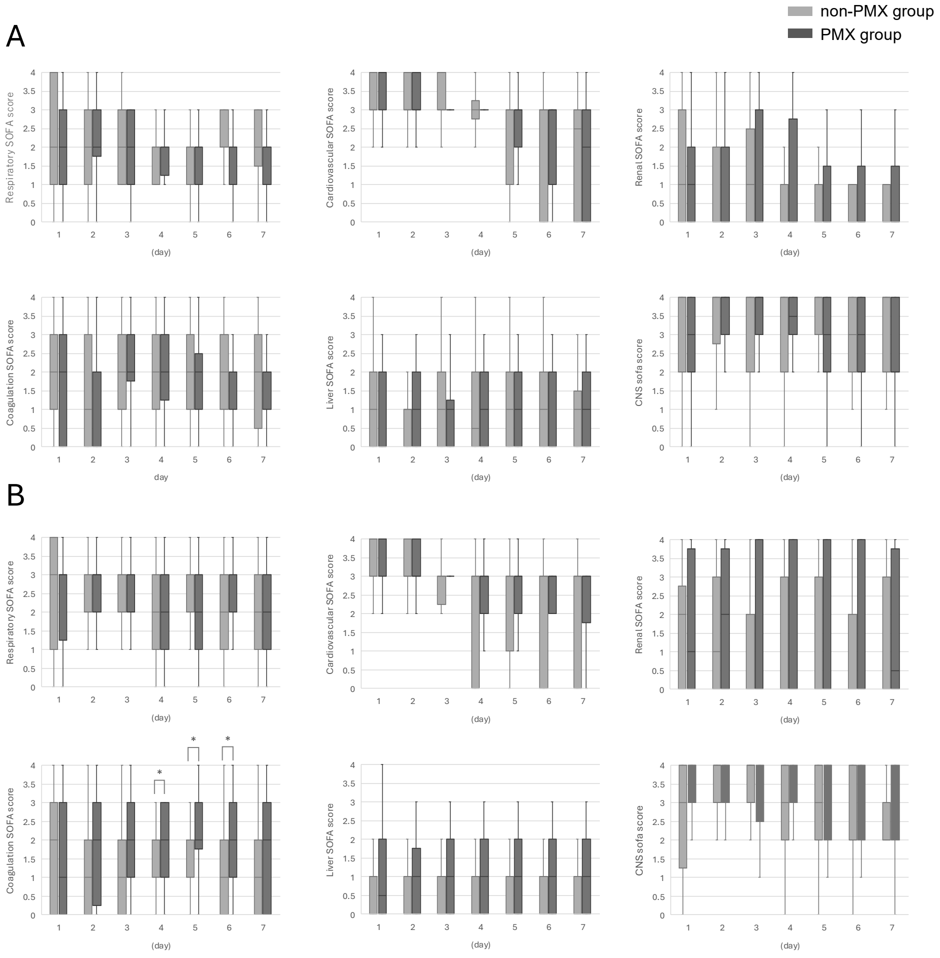

Supplement: Supplementary file 1 — Figure S1: aor70023‐sup‐0001‐FigureS1.pdf. [file AOR-50-271-s003.pdf]
